# Quality of Life Across the Anorexia Nervosa Spectrum: A Comparative Study of Current, Weight‐Restored, and Healthy Individuals

**DOI:** 10.1002/eat.24506

**Published:** 2025-07-18

**Authors:** Stephanie Miles, Erica Neill, Andrea Phillipou

**Affiliations:** ^1^ Centre for Youth Mental Health The University of Melbourne Melbourne Australia; ^2^ Orygen Melbourne Australia; ^3^ Department of Psychology Swinburne University of Technology Melbourne Australia; ^4^ Orygen Specialist Program, Royal Melbourne Hospital Melbourne Australia; ^5^ Department of Mental Health St Vincent's Hospital Melbourne Australia; ^6^ Department of Mental Health Austin Hospital Melbourne Australia

**Keywords:** anorexia nervosa, eating disorders, illness states, quality of life, wellbeing, women

## Abstract

**Background:**

Anorexia nervosa (AN) is a serious mental illness that can greatly impact quality of life (QoL). While research suggests that health‐related QoL is poor in current AN, limited studies have examined QoL in those recovering from AN. This study aimed to investigate health‐related QoL in people with current AN, those weight‐restored from AN, and healthy controls (HCs).

**Methods:**

Participants were 15 women with current AN, 12 weight‐restored women, and 15 HC women. Health‐related QoL was assessed using the Assessment of Quality of Life (AQoL‐8D). Group differences were analyzed using analyses of variance with a priori contrasts.

**Results:**

The current AN group scored significantly lower than the weight‐restored AN and HC groups on the AQoL‐8D subscales of independent living, mental health, happiness, relationships, and self‐worth. The weight‐restored AN group scored equivalently to HCs in QoL. No group differences were found for the pain subscale.

**Discussion:**

Findings highlight the substantial impact of AN on QoL and the potential for QoL to improve with recovery, which could motivate treatment engagement.

**Summary**

- This research demonstrates the poor quality of life experienced by those with current anorexia nervosa.
- Compared to the healthy population, people with anorexia nervosa scored lower in assessments of self‐worth, happiness, relationships, mental health, and independent living.
- People who were weight‐restored from anorexia nervosa scored equivalently to healthy people on many aspects of quality of life.
- This finding could be leveraged in clinical practice to increase motivation in those with current anorexia nervosa and build hope.

## 1 Introduction

Anorexia nervosa (AN) is the most deadly of all mental health diagnoses, affecting up to 3.6% of the population (Arcelus et al. 2011; van Eeden et al. 2021). The physical and mental symptoms associated with AN have a notable impact on quality of life (Jenkins et al. 2011). Quality of life (QoL) refers to someone's subjective view of their life in relation to their goals, standards, and expectations within the context of their culture (The WHOQOL Group 1995). QoL incorporates one's independence, physical health, mental health, social relationships, environment, and religious/spiritual beliefs (The WHOQOL Group 1995). Health‐related quality of life (HRQoL), specifically, is the subjective evaluation of how an illness and its treatment impact one's own physical, psychological, and social functioning (Revicki et al. 2014). Research suggests HRQoL is poor in individuals with AN (Abbate‐Daga et al. 2014; Hovrud and De Young 2015; Neipp et al. 2024; Pohjolainen et al. 2016; Tan et al. 2022), with a recent systematic review reporting that people with AN experience significantly poorer HRQoL than the general population (Ágh et al. 2016).

Limited research has been conducted on QoL for those who are recovering from AN. A large study investigated daily functioning and life engagement in 130 female outpatients with AN (Duriez et al. 2021). Duriez et al. (2021) found that after four months of treatment, participants significantly improved in QoL and dysfunction had decreased. Other work by Abbate‐Daga et al. (2014) found that HRQoL significantly improved in people with AN between emergency hospital admission and discharge (mean admission time = 26 days). In each of these studies, although participant body mass index (BMI) had increased between admission and follow‐up, it nonetheless remained under 18, and participants were not yet weight‐restored. Contrastingly, another study found no differences in social functioning and QoL between people with current AN and those who were weight‐restored (Meguerditchian et al. 2009). Further, a number of studies have reported that BMI was not significantly associated with general and health‐related QoL in AN (Mason et al. 2018; Neipp et al. 2024; Tan et al. 2022). An 8‐year follow‐up study found that patients with AN improved across several dimensions of HRQoL compared to baseline scores at treatment admission (Pohjolainen et al. 2016). Despite these improvements, AN patients were nonetheless notably impaired in some dimensions of HRQoL (e.g., distress, sleep) and HRQoL was significantly poorer than a comparative age‐ and gender‐matched general population (Pohjolainen et al. 2016). Evidently, research is needed to further investigate HRQoL in people with AN across various illness stages. Knowledge of aspects of HRQoL which are most impaired could be used to inform treatment targets and build specific adjunct treatment interventions for those with current AN. Understanding HRQoL in people who are weight‐restored could inform conceptualization about recovery, highlight areas where ongoing support may be needed, and be useful in clinical practice when developing expectations and building motivation for change.

This study aimed to investigate HRQoL in people with current AN and people with a history of AN who were weight‐restored but not fully recovered. It was hypothesized that HRQoL would be significantly poorer in those with current AN compared to HCs. Given the limited literature on QoL in people who are weight‐restored from AN, no explicit hypotheses were developed for this group. Rather, we sought to conduct exploratory analyses that compared weight‐restored participants to the other groups.

## 2 Methods

This paper presents data from a dataset that has been previously analyzed for another study on cognitive flexibility, perfectionism, and rumination in AN (Miles et al. 2023). The methods are described briefly below; however, the original publication should be consulted for further details. The original analysis and publication did not examine QoL.

### 2.1 Participants

Participants in this study were 15 women with a current diagnosis of AN (M age = 26.59, SD = 7.70), 11 women who had a past diagnosis of AN and were currently weight‐restored (AN‐WR; M age = 20.70, SD = 1.89) and 15 HC women (M age = 25.37, SD = 4.28), see Table 1 for details. Inclusion criteria were: female sex, ≥ 18 years old, fluent in English, no history of a head injury or a neurological condition (e.g., autism spectrum disorder), no history of psychosis or psychotic symptoms, and no family history of psychotic disorders. In addition, participants in the current AN group were required to have a current primary diagnosis of AN as determined by a psychologist or psychiatrist and a BMI < 18.5 within the past 3 months. Participants in the AN‐WR group were required to have a previous diagnosis of AN and to have maintained a BMI ≥ 18.5 for a minimum of 3 months. It was intended that the AN‐WR group would not necessarily be fully recovered and free from all behavioral or psychological symptoms of AN, but have achieved weight restoration. Indeed, eight of the 11 AN‐WR participants endorsed behavioral symptoms (binge eating, purging, or fasting) and five participants reported psychological symptoms within the past month. As seen in Table 1, Eating Disorders Examination‐Questionnaire (EDE‐Q) global scores were significantly higher in the AN‐WR group than in HCs. Nonetheless, the EDE‐Q scores for six of the AN‐WR participants fell within one standard deviation of age‐matched community norms (Mond et al. 2006). In line with the Bardone‐Cone et al. (2010) criteria for recovery from an eating disorder, only three participants met criteria for full recovery from AN. Please see the SI for details regarding the current treatments being undertaken by the AN and AN‐WR participants. Due to the poor consensus in the literature regarding the effects of psychiatric medication or comorbid diagnoses on cognitive performance (Lopez et al. 2008), participants in the AN and AN‐WR groups were not excluded if they were taking psychiatric medication or had a diagnosis of another mental illness (other than a psychotic disorder). HCs were required to have no personal or family history of an eating disorder and were excluded if they were currently taking psychiatric medication or had a current formal diagnosis of a mental illness. HCs were not excluded if they had a past diagnosis of a mental illness.

**TABLE 1:** Sample characteristics.

	Group			Test statistics			
	AN	AN‐WR	HC	*F*	*p*	Significant group differences	Effect size
Age	26.59 (7.70)	20.70 (1.89)	25.37 (4.28)	4.00	0.027	AN = HC;AN‐WR < AN, HC	0.17^a^
Current BMI	16.49 (2.01)	22.68 (2.12)	23.52 (2.15)	48.99	< 0.001	AN < AN‐WR, HC; AN‐WR = HC	0.72^a^
Lowest BMI in the past 3 months	15.30 (2.05)	22.26 (2.43)	22.86 (2.37)	49.00	< 0.001	AN < AN‐WR, HC; AN‐WR = HC	0.73^a^
EDE‐Q global score	4.15 (1.03)	2.44 (1.60)	0.85 (1.09)	27.10	< 0.001	AN > AN‐WR, HC; AN‐WR > HC	0.59^a^
				*t*	*p*		
Age of AN onset	15.73 (4.60)	14.10 (2.13)	—	1.20	0.245	—	0.43^b^
Illness duration	10.41 (8.46)	4.00 (2.59)	—	2.31	0.030	AN > AN‐WR	0.94^b^
Duration of weight restoration	—	2.13 (1.74)	—	—	—	—	—

*Note:* Age, age of AN onset, duration of illness, and duration of weight restoration are reported in years.

Abbreviations: AN = anorexia nervosa; AN‐WR = anorexia nervosa, weight‐restored; BMI = body mass index, kg/m^2^; EDE‐Q = Eating Disorders Examination‐Questionnaire; HC = healthy control.

^a^ Eta‐squared effect size.

^b^ Cohen's *d* effect size.

### 2.2 Procedure and Measures

The study was approved by the Swinburne University of Technology human research ethics committee (Ref: 20211127‐8734). Participants were recruited from an eating disorder participant registry, public posters, social media posts, and advertisements shared by Australian eating disorder organizations. All posts and adverts indicated that women with current AN, women who had recovered from AN, and healthy women were eligible to participate in the research. Participation was voluntary, and all participants provided written informed consent. After an initial screening interview via email or phone call, participants attended a face‐to‐face testing session that took approximately an hour and a half. See the original paper for in depth details of procedures (Miles et al. 2023).

AN diagnosis was confirmed using the MINI International Neuropsychiatric Interview 7.0.2 (Sheehan et al. 1998). Clinical demographics, including AN age of onset, illness duration, duration of weight‐restoration, and BMI (current and lowest within the past 3 months) were collected (see Table 1). AN age of onset and illness duration were calculated from the time of significant symptom onset rather than the time of formal AN diagnosis.

#### 2.2.1 Assessment of Quality of Life (AQoL)‐8D (Richardson et al. 2014)

The 35‐item AQoL‐8D was used to evaluate HRQoL across physical and psycho‐social domains. The AQoL‐8D subscales include Independent Living, Pain, Senses, Mental Health, Happiness, Coping, Relationships, and Self Worth. The psychometric scoring algorithm was used and a global psychometric score was calculated to provide an estimate of overall HRQoL. Higher scores are indicative of better QoL. The AQoL‐8D has been demonstrated to be a valid and reliable measure of QoL (Richardson et al. 2014, 2016). For the current study, the Coping and Senses subscales had very poor internal consistency in all three groups (Cronbach's alphas = 0.20–0.67); consequently, these individual subscales were not analyzed (however, they were included in the calculation of the global score as per the scoring algorithm). In the AN‐WR group only, the Mental Health subscale had poor internal consistency (Cronbach's alphas = 0.43), and in the HC group, the Independent Living, Self‐Worth, and Pain subscales had poor internal consistency (Cronbach's alphas = 0.60–0.64). The small sample size may be a contributing factor to the low Cronbach's alphas. Across the three groups, internal consistency was good to excellent for all other subscales (Cronbach's alphas ≥ 0.72).

#### 2.2.2 Eating Disorders Examination‐Questionnaire Version 6.0 (EDE‐Q; Fairburn and Beglin 2008)

The 28‐item EDE‐Q was used to assess the severity and frequency of eating disorder symptomatology over the past 28 days (see Table 1). The EDE‐Q global score was used in the current study as an overall indicator of eating disorder symptoms, with higher scores indicating greater severity/frequency. The EDE‐Q has been demonstrated to have excellent reliability, validity, and consistency (Berg et al. 2012). In the current analyses, internal consistency in all groups was excellent for the EDE‐Q global score (Cronbach's alphas ≥ 0.89).

### 2.3 Statistical Analysis

Analyses were completed using SPSS version 29. Across the EDE‐Q, the data were determined to be normally distributed and there were no univariate outliers within the participant groups. For the AQoL‐8D, the data were determined to be negatively skewed on the Pain subscale within the groups; however, it was deemed unnecessary to undertake any transformations to improve the normality. *t*‐Tests were used to compare the AN and AN‐WR groups on age of onset and illness duration. One‐way between‐groups analyses of variance (ANOVAs) with a priori contrasts (AN and AN‐WR, AN and HC, AN‐WR and HC) were conducted to investigate group differences in AQoL‐8D subscales. Where the assumption of homogeneity of variances was violated, Welch's F is reported. Cohen's *d* effect sizes are reported for the *t*‐tests (where *d* = 0.2 represents a small effect, *d* = 0.5 indicates a medium effect, and *d* = 0.8 is a large effect; Cohen 1988), and *η* ^2^ (where *η* ^2^ = 0.01 represents a small effect, *η* ^2^ = 0.06 indicates a medium effect, and *η* ^2^ = 0.14 is a large effect; Cohen 1988) are reported for the ANOVAs. To control for multiple comparisons, a Bonferroni‐corrected *p*‐value of 0.002 was used for the main analyses. For comparisons of group descriptives (e.g., age, illness duration), a standard *p*‐value of 0.05 was used.

## 3 Results

Significant group differences were found for the AQoL‐8D subscales, and were associated with large effect sizes. As shown in Table 2, the current AN group scored significantly lower than the AN‐WR and HC groups on the Independent Living, Mental Health, Happiness, and Self Worth subscales of the AQoL‐8D. The current AN group also scored significantly lower than the HC group (but not the AN‐WR group) in the Relationships subscale of the AQoL‐8D. In addition, there were no significant differences between the AN‐WR and HC groups for the AQoL‐8D subscales. There were no significant group differences for the Pain subscale.

**TABLE 2:** Group comparisons.

	Group			Test statistics			
	AN	AN‐WR	HC	*F*	*p*	Significant group differences	*η*2
AQoL‐8D							
Independent living	71.85 (17.63)	86.36 (8.74)	92.22 (8.61)	8.03^a^	0.002	AN < HC;AN‐WR = AN, HC	0.35
Pain	74.67 (26.69)	79.09 (30.15)	93.33 (11.13)	3.73^a^	0.43	—	0.12
Mental health	4566. (16.93)	70.80 (9.80)	82.22 (11.62)	23.17^a^	< 0.001	AN < AN‐WR, HC; AN‐WR = HC	0.60
Happiness	35.83 (19.40)	61.36 (13.06)	74.58 (15.21)	21.51	< 0.001	AN < AN‐WR, HC; AN‐WR = HC	0.53
Relationships	57.28 (19.99)	76.09 (12.21)	85.93 (10.49)	13.91	< 0.001	AN < HC;AN‐WR = AN, HC	0.42
Self‐worth	26.67 (23.82)	63.34 (20.16)	83.89 (14.25)	32.14	< 0.001	AN < AN‐WR, HC; AN‐WR = HC	0.63
Global score	53.43 (13.39)	73.76 (7.09)	84.49 (8.62)	34.90	< 0.001	AN < AN‐WR, HC; AN‐WR = HC	0.65

*Note:* Under the ‘Group’ heading, mean scores (standard deviation) are reported.

Abbreviations: AN = anorexia nervosa; AN‐WR = anorexia nervosa, weight‐restored; AQoL‐8D = Assessment of Quality of Life; HC = healthy control.

^a^ Welch's *F* is reported as the assumption of homogeneity of variances was violated.

## 4 Discussion

This study investigated HRQoL in people with AN in current and weight‐restored states. Supporting the primary hypothesis, HRQoL was significantly poorer in women with current AN compared to the other groups, with the exception of the Pain subscale. Women with current AN scored low on mental health, happiness, relationships, self‐worth, and independent living, and effect sizes were very large. This finding supports the findings of other similar research demonstrating that people currently with AN experience poorer QoL than the general population (Abbate‐Daga et al. 2014; Ágh et al. 2016; Hovrud and De Young 2015; Pohjolainen et al. 2016; Tan et al. 2022).

AN‐WR participants scored significantly better in HRQoL compared to participants with current AN and did not significantly differ from HCs in HRQoL, suggesting that after weight restoration and early recovery from AN are achieved, QoL improves across several areas of life. However, as the Mental Health subscale in the AN‐WR group had poor internal consistency, the findings for this subscale should be interpreted with some caution. The findings of this research are in line with the work of Abbate‐Daga et al. (2014) and Duriez et al. (2021) who found that HRQoL improved in people with AN between treatment admission and follow‐up. The findings are also consistent with the work of Meguerditchian et al. (2009) who found no significant differences in social functioning and QoL between current AN participants and AN‐WR participants. In the current study, weight‐restored women did not score significantly differently from women with acute AN or HCs in the Relationships subscale of the AQoL‐8D (scores of 76.09, 57.28 and 85.93, respectively) when the Bonferroni corrected *p*‐value of 0.002 was applied. Given that the obtained *p*‐value for the comparison between these AN and AN‐WR groups was 0.003, a replication of this study with a greater sample size may achieve different results. Nonetheless, despite falling between and not significantly differing from the AN or HC groups, the findings of the current study suggest that weight‐restored women may experience social isolation, difficulties in relationships with families and friends, and social exclusion. Finally, the findings of the current study do not align with the work of Pohjolainen et al. (2016), whereby at 8‐year follow‐up, recovered AN participants scored significantly poorer than an age‐ and gender‐matched general population sample in several domains of HRQoL. Overall, the findings from this research and the broader literature suggest women who have achieved weight restoration from AN experience a QoL that is similar to the general population and notably better than those who are currently ill. As only a handful of studies have investigated QoL in people recovering from AN, further research may be needed to better understand this topic and replicate our findings.

Sy et al. (2013) suggested that the ego‐syntonic nature of AN may persuade people to believe that their lives are better when they are ill, thus creating a rose‐colored glasses effect that discourages recovery from AN. The current research has demonstrated that quality of life is significantly impacted across many different domains in those with current AN. However, the results also indicate that quality of life is significantly improved in those who are recovering from AN; a finding which could be leveraged in clinical practice to increase motivation in those with current AN and build hope.

A notable strength of the current study is that all participants completed comprehensive clinical interviews, and current/past AN status was confirmed. A key limitation of this study is that only women were included, and thus the findings may not generalize to other sexes. It is worth noting, however, that findings in the broader literature are mixed, and there is limited evidence to support sex or gender‐based differences in QoL in people with eating disorders (Dahlgren et al. 2017). Nonetheless, future research on QoL in eating disorders should aim to include participants of all sexes and genders. The sample size was also small, which may have impacted the power of the analyses. Significant differences between the HC and AN‐WR groups in QoL may have remained undetected. It should be noted that the current AN group had a significantly longer illness duration than the AN‐WR group (10.4 years and 4.0 years, respectively). The extended illness duration of the current AN participants—and the participant‐specific factors which may have influenced this extended illness duration—should not be overlooked and may have contributed to the group differences in QoL. Ideally, future research investigating QoL in AN illness and recovery states will match participants on illness duration. Further, this study was cross‐sectional in nature; thus, we cannot establish causality between recovery from AN and improvements in QoL. Future longitudinal research should be conducted to better examine illness trajectories and changes to QoL over time. Another limitation of this work is that a disease‐specific assessment of QoL (such as the Eating Disorders Quality of Life Scale; Adair et al. 2007) was not included. Although the AQoL‐8D assessed HRQoL across physical and psycho‐social domains, a more comprehensive and eating disorder‐specific scale may have enhanced the findings. A final limitation was that the Coping and Senses subscales of the AQoL‐8D were excluded from the analyses due to poor internal consistency. Thus, conclusions cannot be drawn regarding these aspects of QoL.

Overall, the current study found that QoL is poor in current AN but improves in those who are weight restored. This research has potential clinical implications for building motivation in patients with AN.

## Author Contributions

**Stephanie Miles:** conceptualization, formal analysis, data curation, investigation, project administration, writing – review and editing, writing – original draft, methodology. **Erica Neill:** conceptualization, writing – review and editing. **Andrea Phillipou:** conceptualization, writing – review and editing, methodology, supervision.

## Conflicts of Interest

The authors declare no conflicts of interest.

## Supporting information


**Table 1.** Current treatments being undertaken by participants
**Table 2**. Current comorbid diagnoses (as Determined by the MINI International Neuropsychiatric Interview 7.0.2) and of AN and AN‐WR participants.

## Data Availability

Research data are not shared due to ethical restrictions.
