# Supplementary material for: Quality of Life Across the Anorexia Nervosa Spectrum: A Comparative Study of Current, Weight‐Restored, and Healthy Individuals
Source: Int J Eat Disord. 2025 Jul 18;58(10):2009–14. doi: 10.1002/eat.24506 (PMC12501554; doi:10.1002/eat.24506)
Supplement: Supplementary file 1 — Table 1. Current treatments being undertaken by participants Table 2. Current comorbid diagnoses (as Determined by the MINI International Neuropsychiatric Interview 7.0.2) and of AN and AN‐WR participants. [file EAT-58-2009-s001.docx]

# Quality of life across the anorexia nervosa spectrum: A comparative study of current, weight-restored, and healthy individuals

# Supplementary Material

Stephanie Miles^1-3^, Erica Neill^1,2,4^, Andrea Phillipou^1-6^

^1^Centre for Youth Mental Health, The University of Melbourne, Melbourne, VIC, Australia

^2^Orygen, Melbourne, VIC, Australia

^3^Department of Psychology, Swinburne University of Technology, Melbourne, VIC, Australia

^4^Orygen Specialist Program, Royal Melbourne Hospital, Melbourne, VIC, Australia

^5^Department of Mental Health, St Vincent's Hospital, Melbourne, VIC, Australia

^65^Department of Mental Health, Austin Hospital, Melbourne, VIC, Australia

Correspondence details: Dr Stephanie Miles, Orygen, 35 Poplar Rd, Parkville, VIC 3052, Australia. e: [stephanie.miles1@unimelb.edu.au](mailto:stephanie.miles@orygen.org.au).

Table 1: Current treatments being undertaken by participants

|  | **AN** (*n* = 15) | **AN-WR** (*n* = 11) |
| --- | --- | --- |
| **Inpatient** | 1 (6.7%) | 0 |
| **Day patient** | 0 | 0 |
| **Outpatient** | 4 (26.7%) | 0 |
| **Private psychiatrist** | 6 (40%) | 1 (9.1%) |
| **Private psychologist** | 5 (33.3%) | 0 |
| **General Practitioner** | 9 (60%) | 2 (18.2%) |
| **Dietitian** | 2 (13.3%) | 0 |
| **Other** | 2 (13.3%) | 0 |
| **None** | 0 | 9 (81.8%) |

*Note*: Some participants were undertaking multiple treatments at the same time; *n* and percentage of sample are reported. AN = anorexia nervosa; AN-WR = anorexia nervosa, weight-restored.

Table 2: Current comorbid diagnoses (as Determined by the MINI International Neuropsychiatric Interview 7.0.2) and of AN and AN-WR participants

|  | **AN** (*n* = 15) | **AN-WR** (*n* = 11) |
| --- | --- | --- |
| Agoraphobia | 6 | 1 |
| Alcohol use disorder | 3 | 0 |
| Antisocial personality disorder | 1 | 0 |
| Bipolar I | 1 | 0 |
| Bulimia nervosa | 4 | 1 |
| Generalised anxiety disorder | 8 | 2 |
| Major depressive disorder | 5 | 0 |
| Obsessive-compulsive disorder | 3 | 1 |
| Panic disorder | 1 | 1 |
| Post-traumatic stress disorder | 3 | 0 |
| Social anxiety disorder | 6 | 1 |
| Substance use disorder | 1 | 2 |

*Note:* *n* is reported; AN = current anorexia nervosa; AN-WR = anorexia nervosa, weight-restored
